# Rapid Detection of Saxitoxin Using a Nucleic Acid Aptamer Biosensor Based on Graphene Oxide as a Fluorescence Quencher

**DOI:** 10.3390/toxins17090430

**Published:** 2025-08-28

**Authors:** Yi Jiao, Liqing Yang, Junping Hao, Yuhang Wen, Jianhua Wang, Hengchao E, Zhiyong Zhao, Yufeng Chen, Xianli Yang

**Affiliations:** 1 College of Chemistry and Chemical Engineering, Mudanjiang Normal University, Mudanjiang 157012, China; jiaoyimdjnu@163.com (Y.J.); hhhaojunping@163.com (J.H.); 2 Institute for Agro-Food Standards and Testing Technology, Shanghai Academy of Agricultural Sciences, Shanghai 201403, China; wangjianhua@saas.sh.cn (J.W.); ehengchao@saas.sh.cn (H.E.); zhaozhiyong@saas.sh.cn (Z.Z.); 3 State Key Laboratory of Ultrafast Optical Science and Technology, Xi’an Institute of Optics and Precision Mechanics, Chinese Academy of Sciences, Xi’an 710119, China; yangliqing@opt.ac.cn; 4 School of Science, Inner Mongolia University of Science and Technology, Baotou 014010, China; 2023023043@stu.imust.edu.cn

**Keywords:** saxitoxin, aptamer sensor, rapid detection, graphene oxide

## Abstract

Saxitoxin (STX) is a toxin with paralyzing and lethal properties, necessitating the development of a simple analytical method. This study developed a nucleic acid aptamer biosensor using graphene oxide (GO) as a fluorescence quencher for STX detection. GO was combined with M30-f, an STX nucleic acid aptamer modification with 5-carboxyfluorescein, which can produce fluorescence absorption under the conditions of an excitation wavelength of 408 nm and emission wavelength of 515 nm. Based on the principle of fluorescence resonance energy transfer, the fluorescence of M30-f was quenched. In the presence of STX, M30-f specifically binds to STX and dissociates from the GO surface, thereby restoring fluorescence. The STX content can be quantitatively detected through differences in fluorescence absorption. The influence of ultrasonic time on the fluorescence quenching ability of GO was investigated. The aqueous solution of graphene oxide, 30GO, optimized by ultrasound treatment for a duration of 30 min, demonstrated excellent fluorescence quenching capability. 30GO was analyzed utilizing various characterization techniques, including SEM, FT-IR, UV, XPS, XRD, AFM, and contact angle measurements. The methodological validation showed that the established STX sensor exhibits excellent linearity within a concentration range of 10–100,000 ng/L, with a limit of detection (LOD) as low as 0.098 μg/L. In addition, the results further demonstrated the sensor’s high specificity for detecting neurotoxic shellfish toxin STX. The recovery rate for clam samples ranged from 89.12% to 104.71%, while that for oyster samples ranged from 91.20% to 109.65%, with relative standard deviations (RSDs) all below 3%. This aptamer sensor is characterized by its simplicity, high sensitivity, and broad detection range, providing significant technical support for advancing marine biotoxin research.

## 1. Introduction

Saxitoxin (STX) is a highly toxic neurotoxin of paralytic shellfish toxins (PSTs) [1,2,3]. The toxin primarily originates from methanogens in marine settings and cyanobacteria in freshwater, and it can accumulate in shellfish, fish, and other organisms [4,5]. This toxin affects the nervous system, leading to symptoms such as nausea, vomiting, unusual oral sensations, visual impairments, muscle paralysis, and myalgia [6,7]. Each year, approximately 2000 cases of PSP are reported worldwide, with a mortality rate of 15% [8]. Consequently, the World Health Organization (WHO) and the European Food Safety Authority (EFSA) have established a safety limit of 80 micrograms of STX per 100 g of shellfish soft tissue [9]. Additionally, the United States Environmental Protection Agency (EPA) has listed STX in Candidate Contaminant List 4 and List 5, and it is also classified as a controlled substance under the Chemical Weapons Convention [10].

Currently, the primary techniques for detecting STX include high-performance liquid chromatography (HPLC), liquid chromatography–mass spectrometry (LC-MS/MS), and immunoassay [11,12,13]. HPLC and LC-MS/MS offer high sensitivity for quantitative detection but involve complex procedures and require skilled operators [14,15]. Immunoassays are advantageous for their straightforward sample preparation, quick results, and lack of need for advanced equipment [16]. However, this method has drawbacks, such as the susceptibility of enzyme-linked immunosorbent assays (ELISAs) to enzyme interference, sensitivity to environmental factors like temperature and pH, and issues with specificity and high cross-reactivity [17]. The nucleic acid aptamer sensor developed in this study has advantages over the aforementioned methods, including simplicity, high sensitivity, no need for specialized operators, and the capability for on-site detection.

The aptamer, which comes from the Latin word “aptus” meaning fit or pair, was initially introduced by Tuerk and Ellington through a unique oligonucleotide screening method known as Systematic Evolution of Exponentially Enriched Ligands (SELEX) [18]. This method allows for the in vitro screening of oligonucleotide fragments that can specifically bind to target molecules, which may include DNA or RNA fragments. The resulting short-stranded DNA or RNA fragments from this process are referred to as nucleic acid aptamers [19,20]. Aptamers exhibit a strong affinity and selectivity for their target molecules. Their advantages, such as the absence of immunogenicity, in vitro screening, quick synthesis, and the ability to undergo various chemical modifications [21,22], have led to a growing interest in their application within analytical chemistry. Numerous aptamers with high affinity and selectivity for small molecules have been identified, including those targeting marine toxins [23], mycotoxins [24], and veterinary drugs [25].

Graphene oxide (GO) is a derivative of graphene that features various oxygen-containing functional groups, including epoxy, carboxyl, and hydroxyl groups, on its surface [26]. This material is both hydrophilic and biocompatible. In comparison to other bursting agents, GO demonstrates superior fluorescence bursting characteristics due to its SP^2^ hybridized regions, making it a popular choice as a fluorescent receptor in fluorescence resonance energy transfer systems [27,28]. Consequently, GO was selected as the bursting agent for this research.

This study aims to design a nucleic acid aptamer biosensor based on fluorescence resonance energy transfer (FRET), which employs GO as a fluorescence quencher for rapid STX detection. In this study, the 5′ terminus of the STX-specific aptamer (APT) was subjected to modification with 5-carboxyfluorescein (FAM). Subsequently, through the ultrasonic dispersion of GO, the study employed the FAM-modified APT as the fluorescent donor and GO as the fluorescent acceptor, leveraging the principle of FRET for the quantitative detection of STX.

## 2. Results and Discussion

### 2.1. Experimental Principle

**Figure 1:**
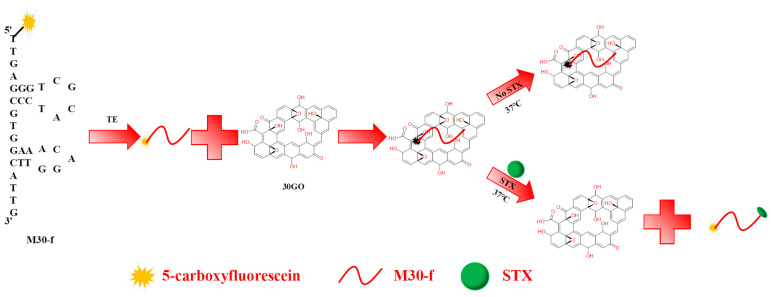
Schematic diagram of STX detection method using FRET-based nucleic acid aptamer sensor.

As illustrated in Figure 1, the oxygen-containing functional groups and conjugated structures on the surface of 30GO enable it to adsorb M30-f through π-π stacking interactions. The fluorescent group at the 5′ end of M30-f is quenched by 30GO via FRET. In the absence of STX, M30-f remains attached to the 30GO surface, preventing fluorescence recovery; thus, STX is not detected at this stage. However, when STX is present, its specific binding with M30-f causes M30-f to detach from the 30GO surface, inhibiting the FRET process and restoring the fluorescence signal of M30-f, which corresponds to the detection of STX.

### 2.2. 30GO Characterisation

#### 2.2.1. SEM Analysis

Figure S1 shows the scanning electron microscope (SEM) image of 30GO, clearly displaying its layered structural characteristics and highlighting the presence of wrinkles and folded areas. These features can be attributed to the formation of sp^3^ hybridized carbon atoms and oxygen functional groups within the basal plane, as well as structural defects in the 30GO material [29,30]. It is this unique structure that endows 30GO with significant adsorption capacity.

#### 2.2.2. FT-IR and UV Analysis

The functional groups on the 30GO surface were analyzed using Fourier transform infrared spectroscopy (FT-IR). The UV–visible absorption spectra of the material in the range of 200–800 nm were recorded. The results are shown in Figure S2.

Figure S2a shows the FTIR diagram of 30GO, which demonstrates distinct characteristic absorption bands: hydroxy-OH (3445.63 cm^−1^), carboxy C=O (1735.14 cm^−1^), aromatic C=C (1635.60 cm^−1^), epoxy C-O (1382.54 cm^−1^), and alkoxy C-OH (1094.41 cm^−1^). The results agree with those reported in the literature [31,32,33]. The findings indicate that oxygen groups on the GO surface carry a negative charge, thereby improving electrostatic adsorption [34]. Epoxy and carbonyl groups disrupt the sp^2^ network, leading to the formation of vacancy defects and the exposure of additional edge adsorption sites [35]. -OH and -COOH groups create hydrophilic regions, facilitating adsorption [36]. Consequently, 30GO exhibits a pronounced adsorption capacity.

According to Figure S2b, 30GO has a maximum absorption peak at 228 nm, which is due to the π-π * leap of atomic C-C bonds, and a shoulder peak at 303 nm, which is due to the n-π * leap of C-O [36,37]. This structure enhances the quenching of fluorescence more effectively.

#### 2.2.3. Contact Angle Analysis

**Figure 2:**
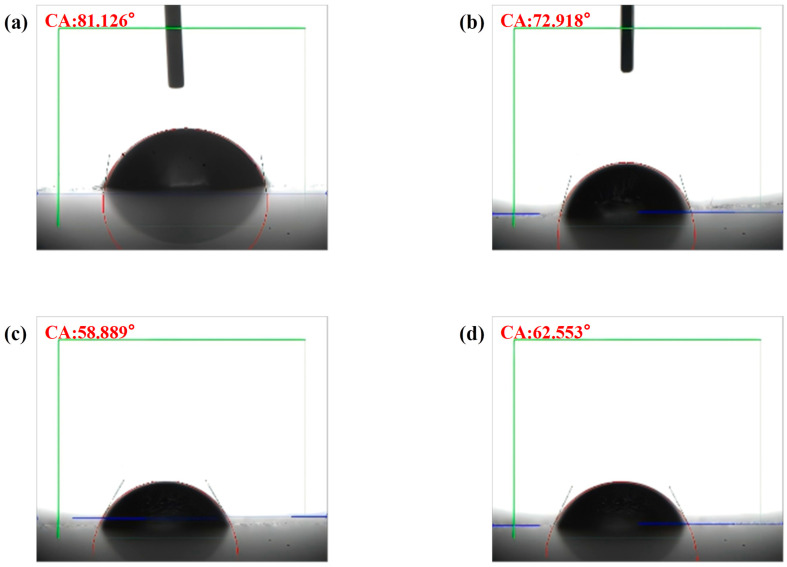
(**a**) The average contact angle of GO samples after 10 min of ultrasonic treatment. (**b**) The average contact angle of GO samples following 20 min of ultrasonic treatment. (**c**) The average contact angle of GO samples subjected to 30 min of ultrasonic treatment. (**d**) The average contact angle of GO samples after 40 min of ultrasonic treatment. The red letters are the average contact angles, the green box indicates the measurement range, the blue lines are the measurement baselines in this figure and the red curve represents the measurement angle.

The contact angle is defined as the angle formed at the junction where the gas, liquid, and solid phases converge, specifically measuring the angle between the tangent to the gas–liquid interface and the solid–liquid interface. Typically, contact angles range from 0° to 90°, which signifies that the material exhibits high solubility in water; conversely, angles between 90° and 180° indicate lower water solubility. As illustrated in Figure 2a–d, the average contact angles (CAs) of GO subjected to ultrasonic treatment for 10, 20, 30, and 40 min are 81.126°, 72.918°, 58.889°, and 62.553°, respectively. These findings demonstrate that the contact angle of GO remains consistently below 90° across all treatment durations. Notably, the contact angle decreases with increasing ultrasonic treatment time, reaching its minimum at 30 min. However, after 40 min of treatment, the contact angle increases, which may be attributed to excessive ultrasonic exposure diminishing the surface hydrophilic functional groups, thereby enhancing the hydrophobic properties of graphene oxide [38,39]. Given that smaller contact angles are associated with higher water solubility, the optimal ultrasonic treatment time for GO was determined to be 30 min. Therefore, 30GO exhibits superior adsorption capacity and fluorescence quenching ability, thereby promoting more effective adsorption of M30-f and quenching of its fluorescent groups.

#### 2.2.4. XRD and AFM Analysis

**Figure 3:**
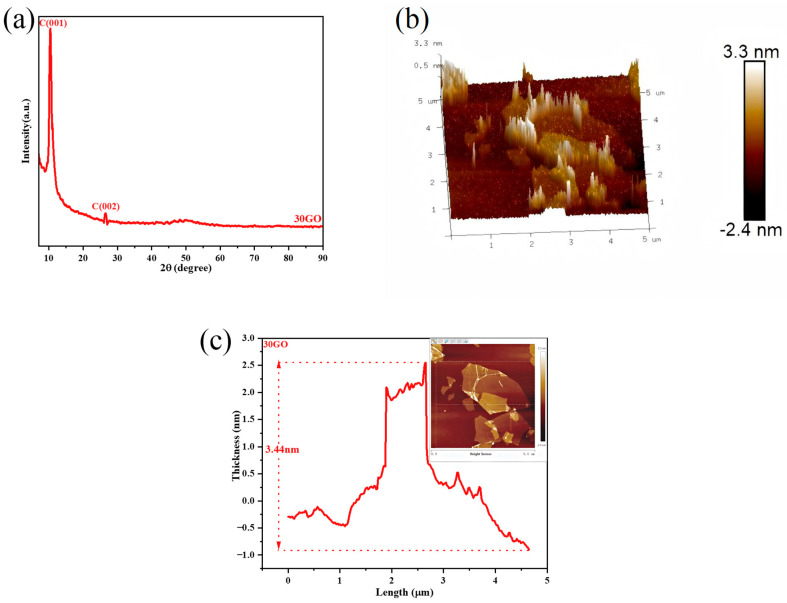
(**a**) XRD pattern of GO sample treated with ultrasound for 30 min. (**b**) Three-dimensional AFM images of 30GO. (**c**) Two-dimensional AFM images of 30GO.

To examine the morphological structure and crystalline phase composition of 30GO, atomic force microscopy (AFM) was utilized for characterization. The physical phase and crystal structure of 30GO were analyzed through X-ray diffraction (XRD), with the findings presented in Figure 3.

Figure 3a presents the X-ray diffraction (XRD) pattern of 30GO. The characteristic C(001) peak of graphene oxide (GO) is observed at 10.46°, while an additional diffraction peak corresponding to C(002) is detected at 26° [40,41]. The interplanar spacing associated with these peaks can be determined by applying Bragg’s equation [42]:

*d* = *λ*/2*sinθ* (1)

where *d* represents the spacing between crystal planes, *λ* denotes the wavelength of the incident X-ray radiation, and *θ* corresponds to the angle of diffraction. The interplanar spacing determined for the C(001) crystal plane is 0.850 nm, whereas for the C(002) crystal plane, it measures 0.340 nm. Due to the larger interlayer spacing of 30GO, the number of adsorption sites is increased. As a result, both the adsorption capacity and fluorescence quenching ability of 30GO are enhanced. Furthermore, 30GO exhibits a better adsorption effect on M30-f and can more effectively quench its surface fluorescent groups.

Figure 3b illustrates that the surface morphology of the 30GO sample is comparatively smooth, exhibiting a roughness value of 0.981 nm. Furthermore, as depicted in Figure 3c, the thickness of the 30GO sample measures 3.04 nm. These results indicate that both the surface roughness and thickness of 30GO are relatively minimal, a feature that is advantageous for improving its fluorescence quenching efficiency and adsorption capacity. Consequently, this promotes the adsorption of M30-f onto the 30GO surface and enhances the quenching of the fluorescent moieties within M30-f.

#### 2.2.5. XPS Analysis

**Figure 4:**
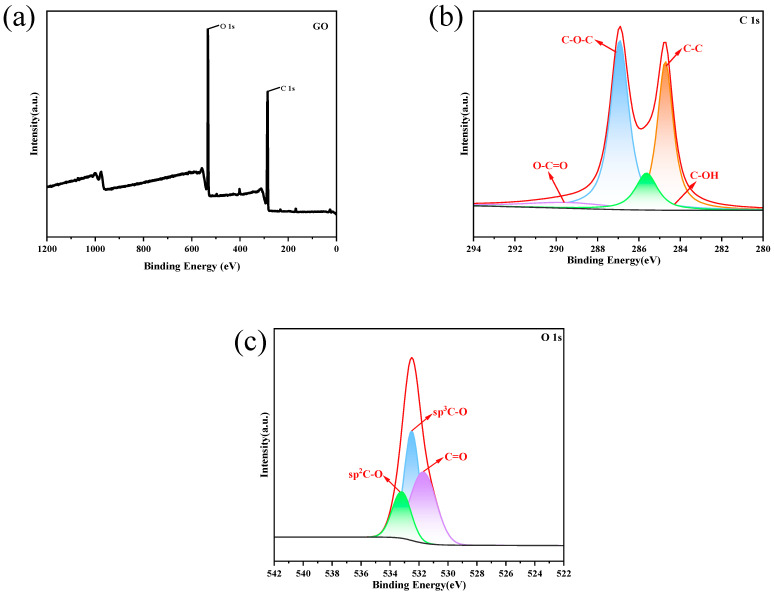
XPS spectra of GO samples treated with ultrasound for 30 min: (**a**) the wide scan spectra, (**b**) the C 1 s core-level spectrum, and (**c**) the O 1 s core-level spectrum.

High-resolution XPS spectra were used to analyze and determine the chemical state of 30GO. All obtained spectra were calibrated against the C 1 s electronic peak at 284.6 eV. The results are shown in Figure 4.

Figure 4a illustrates the full-range XPS spectrum of the 30GO sample. The sample contains C and O elements with distinct photoelectron peaks at binding energies of 531 eV (O 1 s) and 286 eV (C 1 s). The C and O elements are from the original 30GO sample.

The high-resolution XPS spectra of the C 1 s orbitals in 30GO are shown in Figure 4b. The characteristic peaks of GO at binding energies of 284.6 eV (C-C), 285.8 eV (C-OH), 286.9 eV (C-O-C), and 289.1 eV (O-C=O) corresponded to the C atoms, hydroxyls, epoxides, and carboxyls in the sp^2^ graphene lattice, and the C 1 s peaks were analyzed to show that the most functional groups are associated with C-O-C bonds in graphene oxide [43,44,45]. A high-resolution spectrum of O 1 s is shown in Figure 4c. The O 1 s spectrum was fitted to the peaks of C=O (531.7 eV), sp^3^ C-O (532.5 eV), and sp^2^ C-O (533.2 eV), which corresponded to O atoms in the carbonyl, hydroxyl, epoxy, and phenolic groups, respectively [45]. The results of the peak analysis indicate that most of the functional groups in 30GO are associated with sp^3^-type C-O bonds.

The presence of oxygen-containing functional groups in 30GO makes it hydrophilic and adsorptive, the overall analysis of XPS shows that 30GO contains more oxygen-containing functional groups, and the higher the concentration of oxygen-containing functional groups, the better its hydrophilicity and adsorptive property [46,47].

### 2.3. STX Standard Solution Assay

#### 2.3.1. Feasibility Confirmation Studies

**Figure 5:**
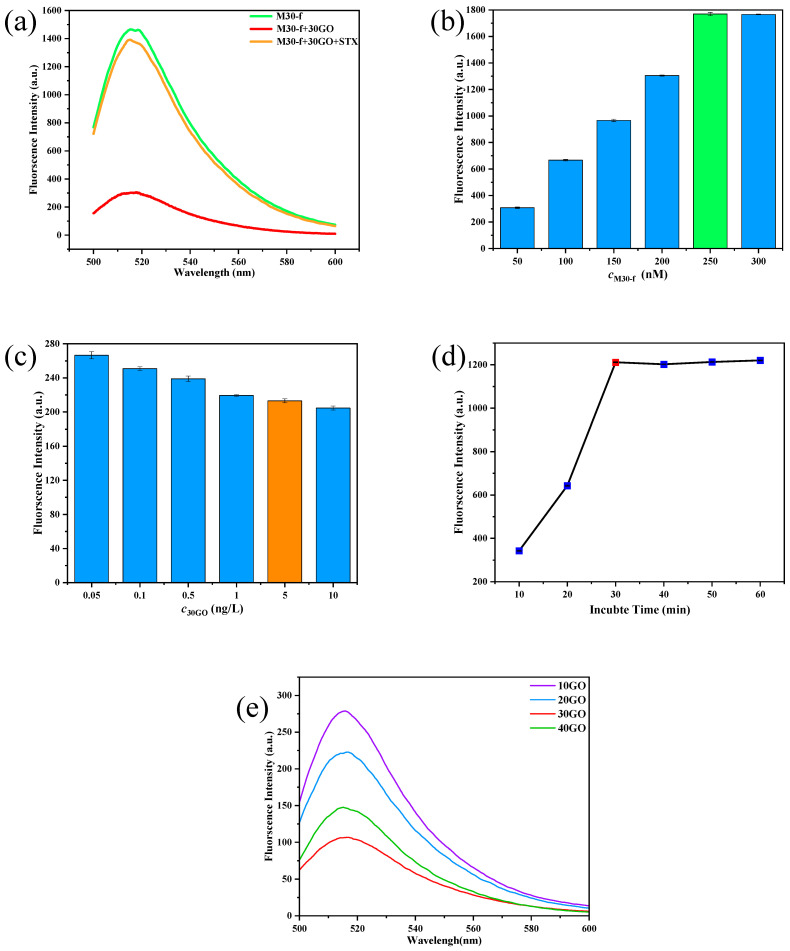
(**a**) Schematic diagram of feasibility of verification experiment. (**b**) Effect of M30-f concentration on fluorescence intensity. (**c**) Selection of 30GO concentration. (**d**) Effect of incubation time on fluorescence intensity. (**e**) The impact of the duration of ultrasonic dispersion of GO on fluorescence intensity.

The experiment’s feasibility was confirmed through an on–off–on method, as illustrated in Figure 5a. Initially, the fluorescence intensity of the aptamer M30-f was recorded. Subsequently, 30GO was introduced, followed by a second measurement of fluorescence intensity, which demonstrated a reduction in fluorescence. Upon the addition of the STX standard solution, an increase in fluorescence intensity was observed. These results align with the underlying experimental principle, thereby validating the feasibility of the experimental approach.

#### 2.3.2. Optimization of Experimental Parameters

To ensure the stability of the nucleic acid aptamer sensor’s performance, a thorough optimization of various parameters was undertaken. This included the concentration of M30-f, the concentration of 30GO, the incubation duration, and the ultrasonic dispersion time of GO. The results of these experiments are illustrated in Figure 5b–e.

Figure 5b shows the results of optimal concentration selection of M30-f. When the final concentration of M30-f was 250 nM, the maximum fluorescence intensity was obtained at 515 nm; when the final concentration was 300 nM, the fluorescence intensity was basically unchanged, and therefore 250 nM was chosen as the final concentration of M30-f.

An excessive concentration of 30GO would hinder M30-f’s ability to specifically bind to STX, preventing fluorescence recovery, while too low a concentration of 30GO would fail to fully burst M30-f, leading to increased initial fluorescence. As illustrated in Figure 5c, a final 30GO concentration of 10 ng/L resulted in the most effective bursting of M30-f, but fluorescence could not be recovered after STX was added, due to the high concentration of 30GO causing nonspecific adsorption of fluorescent groups. In contrast, at a final 30GO concentration of 5 ng/L, M30-f could still be burst, and fluorescence was recoverable after adding STX. Thus, 5 ng/L was determined to be the optimal 30GO concentration. Figure 5d presents the results for the ideal incubation time, showing that fluorescence intensity increased from 0 to 30 min, indicating that M30-f and STX were not specifically binding during this period. After 30 min, the fluorescence intensity stabilized, suggesting that specific binding had occurred, leading to the conclusion that 30 min was the optimal incubation time for the experiments.

Figure 5e illustrates the outcomes of the ultrasonic dispersion of GO over a specified period. The data indicate that between 10 and 30 min, there is a gradual decrease in fluorescence intensity, suggesting an enhancement in the fluorescence quenching capability of GO as the duration of ultrasonic dispersion increases. However, beyond 30 min of ultrasonic treatment, the fluorescence intensity began to increase. This phenomenon may be attributed to the potential reduction in the quenching ability of GO due to excessive ultrasonic exposure, which could lead to a decrease in the number of surface hydrophilic functional groups. Consequently, this reduction may increase the hydrophobicity of GO and diminish its adsorption capacity for heavy metals [38,39]. Therefore, it can be concluded that the optimal duration for ultrasonic dispersion is 30 min.

#### 2.3.3. Detection of STX Using the Developed Aptamer Sensor

**Figure 6:**
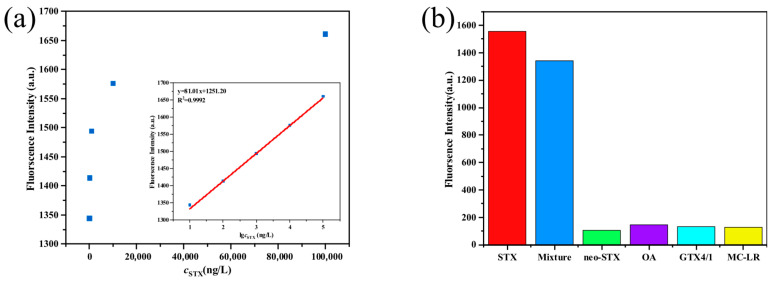
(**a**) Fluorescence intensity at 515 nm as a function of concentration of STX. (**b**) Selectivity of the method with STX, neo-STX, OA, GTX4/1, MC-LR, and the mixture of marine toxins.

Different concentrations of STX were detected under the optimal conditions, and the results are shown in Figure 6a. The standard curve equation was y = 81.01x + 1251.20 with the correlation coefficient of R^2^ = 0.9992, which showed a good linear relationship between the logarithm of STX concentration and fluorescence intensity in the range of 10 ng/L to 10^5^ ng/L, and the LOD was 0.098 μg/L. This value was derived by applying the standard deviation of 2.40 from the blank sample and the slope of 81.01 obtained from the calibration curve into the LOD calculation formula (3.3 × *σ/S*). In addition, the LOD of the sensitivity sensors developed in this study was lower than most of the reported STX detection methods, and the linear detection range was relatively wide, as shown in Table 1.

#### 2.3.4. Selectivity Test

To assess the high selectivity of the nucleic acid aptamer sensor for STX, this study employed a mixture comprising neosaxitoxin (neo-STX), gonyautoxins 4 and 1 (GTX4/1), okadaic acid (OA), and microcystin-LR (MC-LR) as test analytes (Figure S3). Although marine organisms produce a diverse array of toxins, STX constitutes only approximately 5% of the total toxicity but remains the most potent and significant neurotoxic shellfish toxin. Among paralytic shellfish toxins, GTX4/1 and neo-STX are neurotoxins structurally analogous to STX. OA serves as a representative toxin responsible for diarrhetic shellfish poisoning (DSP), while MC-LR is the most prevalent toxin found in marine environments. Consequently, neosaxitoxin, gonyautoxins 4 and 1, okadaic acid, and microcystin-LR were selected for specificity testing. The results of the specificity assessment are presented in Figure 6b. The fluorescence intensities corresponding to neo-STX, GTX4/1, OA, and MC-LR were observed to be minimal. Furthermore, in the analysis of a mixture containing five toxins, STX was detected rapidly and with high accuracy, exhibiting a fluorescence intensity markedly greater than that of the other toxins. Notably, when STX was the sole analyte present, the fluorescence intensity reached its maximum value, demonstrating that the sensor possesses excellent specificity and selectivity toward STX.

### 2.4. Actual Sample Test

To validate the capability of the nucleic acid aptamer sensor for detecting saxitoxin in authentic samples, shellfish specimens were analyzed following spiking with a standard saxitoxin (STX) solution. The Asian hard clam, a benthic filter feeder, readily accumulates toxins from sedimentary environments, whereas the razor clam predominantly resides in tidal flats and is directly exposed to waters affected by red tides. Given their respective habitats and feeding behaviors, both species are susceptible to STX contamination. Consequently, samples of Asian hard clam and razor clam were chosen for this evaluation. The findings are presented in Table 2. The recovery rates for the clam samples varied between 89.12% and 104.71%, with relative standard deviations (RSDs) under 3%. Similarly, the recovery rates for the razor clam samples ranged from 91.20% to 109.65%, also with RSDs below 3%. These results suggest that the sensor is effective for detecting STX in actual samples.

## 3. Conclusions

In this study, we developed a rapid detection approach for STX employing a nucleic acid aptamer-based biosensor utilizing FRET. Our investigation demonstrated that the binding affinity of GO toward M30-f, an STX-specific aptamer, was significantly influenced by the duration of ultrasonic pretreatment applied to GO. Optimization experiments indicated that GO subjected to 30 min of ultrasonic treatment exhibited the highest aptamer adsorption capacity and optimal fluorescence recovery performance. Additionally, an initial GO concentration of 5 ng/L was identified as most effective for fluorescence quenching. The fluorescence intensity peaked at an incubation temperature of 37 °C. This method achieved an LOD of 0.098 µg/L and displayed a strong linear correlation within the STX concentration range of 10 to 10^5^ ng/L (R^2^ = 0.9992). In spiked real-sample analyses, the sensor accurately quantified STX, with recovery rates ranging from 89.12% to 109.65%. The biosensor exhibited high selectivity and specificity toward STX in the presence of potential interfering substances. Overall, this detection strategy satisfies the criteria for reliable STX monitoring and offers a valuable framework for the rapid detection of other small-molecule toxins and analytes.

## 4. Materials and Methods

### 4.1. Materials

Saxitoxin (GR) was purchased from Qingdao Pribolab Biotech Co., Ltd. (Shandong, China). Graphene oxide was obtained from Shanghai HanLang New Material Technology Co., Ltd. (Shanghai, China). Tris-EDTA (TE) buffer solution was acquired from Sangon Biotech Co., Ltd. (Shanghai, China). Acetic acid was purchased from Shanghai Aladdin Biochemical Technology Co., Ltd. (Shanghai, China). The M30-f used in this study was produced at Sangon Biotech Co., Ltd. (Shanghai, China). The sequences were as follows: FAM-M30-f (5′-FAM-TTGAGGGTCGCATCCCGTGGAAACAGGTTCATT G-3′).

### 4.2. Ultrasonic Dispersion of GO

An accurately measured quantity of 12.5 mg of graphene oxide powder was initially weighed and dissolved in ultrapure water. The resulting solution was then diluted to a final volume of 50 mL, yielding a concentration of 250 mg per liter. Subsequently, the graphene oxide solution underwent dispersion using an ultrasonic cell disruptor (JingXin, Shanghai, China) operated at 80% power. The sonication was performed for durations of 10, 20, 30, and 40 min, respectively, with the resulting samples designated as 10GO, 20GO, 30GO, and 40GO.

### 4.3. Material Characterization

The crystal structure of 30GO was analyzed utilizing an X-ray diffractometer (Bruker D8 ADVANCE, Germany), which operated within a 2θ scanning range of 20–90° and employed a scanning step of 0.02°. The morphological and structural characteristics of the materials were examined using a scanning electron microscope (HITACHI, Tokyo Metropolis, Japan) and an atomic force microscope (Park Systems, Suwon, Republic of Korea). The hydrophilicity of the materials was assessed through measurements taken with a contact goniophotometer (SINDIN, Dongguan, China). X-ray photoelectron spectroscopy (XPS) analyses were conducted using an Escalab 250Xi spectrometer (ThermoFisher, MA, USA). Infrared spectra were obtained with a Nicolet iS5 Fourier Transform Infrared Spectrometer (Thermo Scientific, WI, USA). Additionally, UV–visible absorption spectra within the wavelength range of 200–800 nm were recorded using a Shimadzu UV 3600plus spectrophotometer (Shimadzu, Japan).

### 4.4. STX Standard Solution Test

The optimal concentrations of M30-f and 30GO were added to a 1.5 mL centrifuge tube, and the mixture of 30GO and M30-f was vortexed and shaken for 1 min to quench the fluorescence. The final concentrations of 10 ng/L, 100 ng/L, 1000 ng/L, 10,000 ng/L, and 100,000 ng/L of STX standard solution were added, and the mixture was diluted to 1 mL with TE buffer, vortexed for 1 min, and then incubated at 37 °C for 30 min. Finally, the fluorescence spectrometer (HITACHI, Tokyo Metropolis, Japan) parameters were set to an excitation wavelength of 408 nm and the fluorescence intensity of all centrifuge tube solutions was recorded at the emission wavelength of 515 nm. A standard curve was plotted using the logarithm of the STX concentration as the horizontal axis and F-F_0_ as the vertical axis, and the detection limit was calculated according to the formula below [54]:

*LOD* = 3.3*σ*/*S* (2)

where *σ* represents the standard deviation of the blank group, while *S* denotes the slope of the calibration curve.

### 4.5. Specificity Test

Initially, optimal concentrations of M30-f and 30GO were added separately to six 1.5 mL centrifuge tubes, followed by vortexing for one minute to ensure complete fluorescence quenching. Subsequently, each tube received 10 μg/L of neo-STX, OA, GTX4/1, MC-LR, the STX-containing mixed toxin, and STX, respectively. The solutions were then diluted to a final volume of 1 mL with TE buffer, vortexed for one minute, and incubated at 37 °C for 30 min. Finally, fluorescence measurements were conducted using a fluorescence spectrometer set to an excitation wavelength of 408 nm, and the emission intensity at the emission wavelength of 515 nm was recorded for all samples.

### 4.6. Pre-Treatment of Clam and Razor Clam Samples

Specimens of clams and razor clams were purchased from the agricultural market located in Fengxian District, Shanghai. Initially, the soft tissues were carefully separated from the shells of the shellfish samples. These tissues were then thoroughly rinsed and homogenized using a homogenizer. A 2.5 g aliquot of the homogenized tissue was transferred into a 10 mL centrifuge tube, to which 100 µg/L of STX standard solution was added. The mixture was vortexed for 1 min to ensure complete mixing, followed by incubation at 37 °C for 30 min. Subsequently, 7.5 mL of 1% acetic acid aqueous solution was introduced into the centrifuge tube and vortexed for an additional minute to achieve thorough blending. The tube was then subjected to ultrasonic extraction at ambient temperature with full power (100%) for 30 min in an ultrasonic bath to facilitate STX extraction from the shellfish tissues. After extraction, the sample was centrifuged at 4 °C and 5000 rpm for 10 min. The supernatant was carefully collected and filtered three times through a 0.22 µm membrane filter. The final filtrate was stored at 4 °C for further analytical procedures.

## Figures and Tables

**Table 1:** Comparison of the aptasensor and the other reported methods.

Aptasensors	Linear Detection Range		LOD		Literature Resources
	Reported Range	Converted Range (nM)	Reported LOD	Converted LOD (nM)	
Electrochemical aptasensor	0.9–30 nM	0.9–30	0.38 nM	0.38	[48]
Electrochemical aptasensor	1–1000 nM	1–1000	1 nM	1	[49]
Fluorescent aptasensor	0–24 ng/mL	0–64.48	1.8 ng/mL	4.84	[50]
Fluorescence aptasensor	1–5000 nM	1–5000	0.6 nM	0.6	[51]
SERS sensor	10–200 nM	10–200	11.7 nM	11.7	[52]
LSPR-based aptasensor	5–10,000 μg/L	16.72–33,440	2.46 μg/L	8.23	[53]
Fluorescent aptasensor	10–100,000 ng/L	2.7 × 10^−2^–268.7	0.098 μg/L	0.26	This work

**Table 2:** This method was used to detect STX in real samples (n = 3).

Specimen	Addition Level (μg/kg)	Recovery (%, Mean)	RSD (%)
Manila clam	1	89.12	2.83
	10	97.72	2.13
	100	104.71	1.65
Razor clam	1	109.65	1.40
	10	102.33	2.59
	100	91.20	2.91

## Data Availability

The original contributions presented in this study are included in the article and Supplementary Materials. Further inquiries can be directed to the corresponding authors.

## Supplementary Materials

The following supporting information can be downloaded at: https://www.mdpi.com/article/10.3390/toxins17090430/s1. Figure S1: SEM images of GO samples treated with ultrasound for 30 min. Figure S2: (a) FT-IR of GO sample treated with ultrasound for 30 min. (b) UV-Vis absorption spectra of GO after 30 min of ultrasonic treatment. Figure S3: (a) Chemical structure of STX. (b) Chemical structure of neo-STX. (c) Chemical structure of GTX4/1. (d) Chemical structure of OA. (e) Chemical structure of MC-LR.
