# Supplementary material for: Rapid Detection of Saxitoxin Using a Nucleic Acid Aptamer Biosensor Based on Graphene Oxide as a Fluorescence Quencher
Source: Toxins (Basel). 2025 Aug 28;17(9):430. doi: 10.3390/toxins17090430 (PMC12474413; doi:10.3390/toxins17090430)
Supplement: Supplementary file 1 [file toxins-17-00430-s001.zip › toxins-3821252-supplementary.pdf]

toxins

MDPI

Rapid detection of saxitoxin using a nucleic acid aptamer biosensor based on graphene oxide as a fluorescence quencher

Yi Jiao [1,2,†], Liqing Yang [3,†], Junping Hao [1,2], Yuhang Wen [4], Jianhua Wang [2], Hengchao E [2], Zhiyong Zhao [2], Yufeng Chen [1,*] and Xianli Yang [1,2,4,*]

[1] College of Chemistry and Chemical Engineering, Mudanjiang Normal University, Mudanjiang 157012, China; jiaoyimdjnu@163.com (Y.J.); hhhaojunping@163.com (J.H.)
[2] Institute for Agro-Food Standards and Testing Technology, Shanghai Academy of Agricultural Sciences, Shanghai 201403, China; wangjianhua@saas.sh.cn (J.W.); ehengchao@saas.sh.cn (H.E.); zhaozhiyong@saas.sh.cn (Z.Z.)
[3] State Key Laboratory of Ultrafast Optical Science and Technology, Xi'an Institute of Optics and Precision Mechanics, Chinese Academy of Sciences, Xi'an 710119, China; yangliqing@opt.ac.cn (L.Y.)
[4] School of Science, Inner Mongolia University of Science and Technology, Baotou 014010, China; 2023023043@stu.imust.edu.cn (Y.W.)
* Correspondence: 1002014@mdjnu.edu.cn (Y.C.); yangxianli@saas.sh.cn (X.Y.)
† These authors contributed equally to this work.

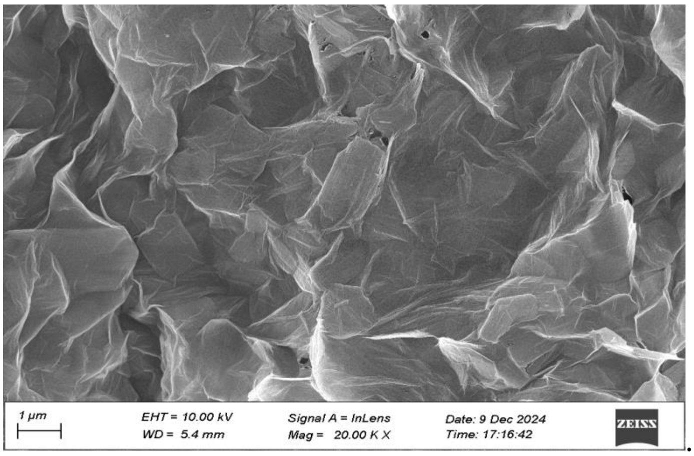

Figure S1. SEM images of GO samples treated with ultrasound for 30 mintures.

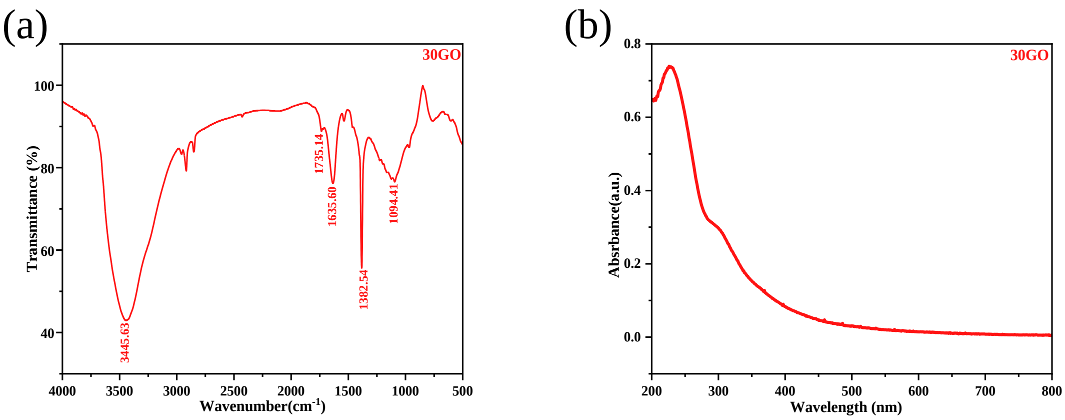

Figure S2. (a) FT-IR of GO sample treated with ultrasound for 30 minutes. **(b)** UV-Vis absorption spectra of GO after 30 minutes of ultrasonic treatment.

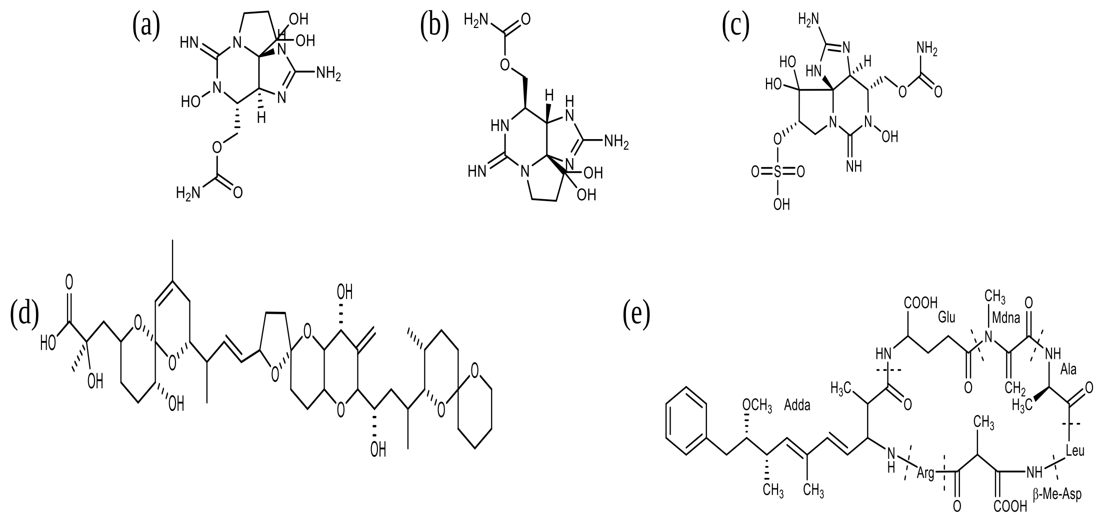

Figure S3. (a) Chemical structure of STX. **(b)** Chemical structure of neo-STX. **(c)** Chemical structure of GTX4/1. **(d)** Chemical structure of OA. **(e)** Chemical structure of MC-LR.
